# A Survey of Emerging DDoS Threats in New Power Systems

**DOI:** 10.3390/s26041097

**Published:** 2026-02-08

**Authors:** Fan Luo, Siqin Fan, Guolin Shao

**Affiliations:** 1 College of Information Engineering, Nanchang University, Nanchang 330031, China; w2973076436@163.com (F.L.); 18879143180@163.com (S.F.); 2 School of Software, Nanchang University, Nanchang 330031, China

**Keywords:** Advanced Persistent Threats, Distributed Denial-of-Service, large language models, smart grid

## Abstract

Distributed Denial-of-Service (DDoS) attacks remain the most pervasive and operationally disruptive cyber threat and are routinely weaponized in interstate conflict (e.g., Russia–Ukraine and Stuxnet). Although attack-chain models are standard for Advanced Persistent Threat (APT) analysis, they have seldom been applied to DDoS, which is often framed as a single-step volumetric assault. However, ubiquitous intelligence and ambient connectivity increasingly enable DDoS campaigns to unfold as multi-stage operations rather than isolated floods. In parallel, large language models (LLMs) create new opportunities to strengthen traditional DDoS defenses through richer contextual understanding. Reviewing incidents from 2019 to 2024, we propose a three-phase DDoS attack chain—preparation, development, and execution—that captures contemporary tactics and their dependencies on novel hardware, network architectures, and application protocols. We classify these patterns, contrast them with conventional DDoS, survey current defenses (anycast and scrubbing, BGP Flowspec, programmable data planes, adaptive ML detection, API hardening), and outline research directions in cross-layer telemetry, adversarially robust learning, automated mitigation orchestration, and cooperative takedown.

## 1. Introduction

### 1.1. Motivation

With rapid advancements in information technology—particularly in large language models (LLMs), cloud computing, the Industrial Internet of Things (IIoT), and blockchain—DDoS attacks have not only increased in frequency but also become more sophisticated over the past five years. These technologies, while disruptive and innovative, often harbor vulnerabilities that attackers exploit to devise new DDoS vectors. For example, blockchain-based DoS (BDoS) [1] and LOFT attacks [2] in software-defined networking (SDN) environments demonstrate how new network architectures can be targeted. Additionally, traditional DDoS methods such as flooding and amplification have evolved, leveraging the multiplexing capabilities of HTTP/2 to intensify their impact [3]. The Mlytics Security Operations Center’s report of a suspected AI-driven DDoS attack in 2023 further intensified this trend [4]. Given these developments, the cybersecurity community urgently needs to deepen its understanding of emerging DDoS attacks and develop effective countermeasures.

Current models for analyzing DDoS attacks are often inadequate for addressing the complexity of emerging DDoS campaigns. These models primarily describe the execution phase, which is only one of several activities necessary for a successful operation. Little work to date analyzes the activities behind these attacks—how adversaries prepare, formulate, and execute these strategies. This has limited our ability to identify the challenges posed by complex attacks. To bridge this gap, we propose a DDoS attack-chain framework, inspired by the “kill chain” model traditionally used to dissect complex cyberattacks such as Advanced Persistent Threats (APTs). The kill chain model segments a cyberattack into distinct, non-exclusive phases or layers, providing a detailed view of an attacker’s sequence of actions. Despite its infrequent application to DDoS, which is often perceived as less complex, the increasing sophistication of these attacks makes the kill chain model particularly relevant. By adapting this model to DDoS contexts, our framework addresses not only execution but also the critical preparatory and developmental stages. This enables deeper characterization and earlier identification of emerging DDoS attacks, addressing the limitations of conventional models and enhancing defensive capabilities. Our study rigorously applies this model to DDoS attacks, seeking to answer the question “Have any new types of DDoS attacks emerged following the emergence and development of new technologies such as LLMs?”

To achieve a thorough analysis of the state-of-the-art literature, we investigated approximately 200 papers published in top-tier networking and security venues from 2019 to 2024, including SIGCOMM, NSDI, INFOCOM, IEEE S&P, USENIX Security, NDSS, CCS, and the IEEE Transactions series. These publications, representative of the cutting edge in this field, provide a robust basis for identifying the latest characteristics and trends in emerging DDoS attacks. We identified common patterns and defined a three-phase DDoS attack-chain model, including preparation, development, and execution. (1) Preparation covers botnet acquisition (IoT/OT and SOHO routers), access brokering, and traffic staging. (2) Development encompasses weaponization and orchestration (booter services, reflection/amplification, CDN, and DNS abuse). (3) Execution includes multi-vector floods (carpet bombing), protocol and application-layer attacks (TLS handshake exhaustion, HTTP/2 Rapid Reset, and QUIC), and adaptive evasion. Each phase is crucial to attack success, and its implementation varies with the techniques exploited and vulnerabilities targeted. Based on the attack chain, emerging DDoS attacks are further classified into three categories: device ecosystem-targeted, network architecture-based, and protocol-based. Our attack chain-based analysis helps researchers and academics understand the mechanisms of emerging DDoS attacks and facilitates the evaluation of potential defensive solutions.

### 1.2. Comparison with Existing Surveys

To highlight the key contributions of our survey, Table 1 compares existing DDoS survey articles with our work, focusing on traditional and emerging threats and the corresponding defense mechanisms. The analysis uses criteria such as coverage of attack types, depth of defense analysis, and the introduction of new analytical models. In Table 1, we find that our work makes significant contributions in the following areas.

- Focus on emerging DDoS attacks: This survey investigates attacks arising from new hardware devices, new network architectures, and new applications, which are underrepresented in existing surveys.
- A new attack-chain analysis framework: This survey characterizes the complexity of emerging DDoS attacks, and an attack-chain framework was constructed, offering a fine-grained understanding of attack phases and methodologies.
- Analysis of potentially effective defenses against emerging DDoS attacks: Our survey outlines existing defense strategies that serve as countermeasures to emerging DDoS attacks, establishing a foundation for current and future research.

Therefore, this survey offers a comprehensive and systematic analysis of emerging DDoS attacks, uniquely summarizing the evolving patterns and challenges from an attack-chain perspective. By integrating a broad spectrum of new information technologies, we close significant gaps in the existing literature, particularly in how emerging DDoS attacks are analyzed and understood. Our study is particularly valuable to researchers and practitioners, not only detailing the complexities of current DDoS strategies but also highlighting potential vulnerabilities within modern defense mechanisms. It can guide the development of more robust defense strategies and inspire future research into effective countermeasures against the dynamically evolving landscape of cyber threats.

### 1.3. Scope and Focus of This Survey

To clearly define the scope, we provide a general, attack-chain-based survey of emerging DDoS campaigns across modern cyber–physical infrastructures while using new power systems (smart grids) as a representative and critical case study to ground the discussion and extract transferable defense principles. Our emphasis is on how emerging DDoS capabilities manifest in the power system context after deep integration with enabling technologies (e.g., IoT/IIoT and modern communication infrastructures) and how recent advances—particularly LLM-enabled security analytics and deception-based defense—can support practical countermeasures. Examples beyond power systems are included to identify attack-chain patterns and defense principles that apply to and are informative for new power-system security.

## 2. Preliminaries

### 2.1. DDoS Attack Model

**Figure 1:**
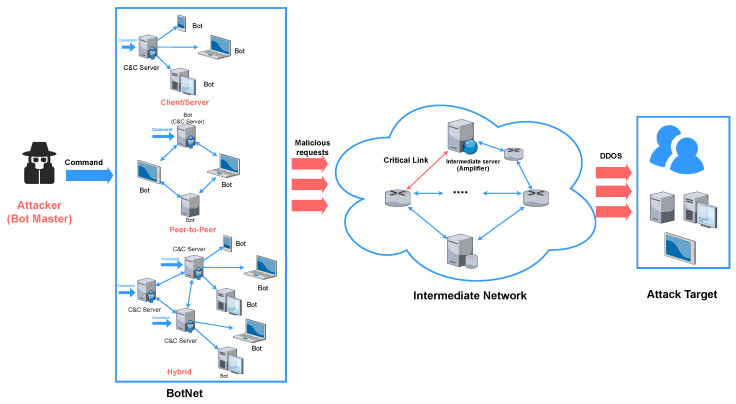
A typical entity-centric DDoS attack model. This baseline view captures the main participants and traffic flow and primarily reflects the execution of a DDoS attack; it motivates our proposed three-phase attack-chain framework (Figure 2) by highlighting that emerging DDoS campaigns also involve substantial profiling and strategy formulation activities beyond the final traffic flood.

According to the current literature, a typical DDoS attack consists of four main components: the attacker, the botnet, the intermediate network, and the attacked target, as in Figure 1.

- Attacker: The attacker, also known as the botnet master, controls a large network of bots, which are compromised, vulnerable devices [13]. By utilizing command and control (C&C) servers, the attacker can remotely manage these bots.
- Botnet: A typical botnet includes both C&C servers and individual bots. Depending on the position of the C&C servers within the botnet, three main communication architectures are employed: client–server, peer-to-peer, and hybrid (Figure 1) [14]. Some services offer botnet rentals, charging based on the duration and intensity of the attack [10].
- Intermediate Network: The intermediary network plays a critical role as an attack vector in a DDoS attack. It consists of key links and intermediate servers that can be exploited to amplify or redirect attack traffic, such as in reflection/amplification attacks [15]. This component is particularly crucial in amplification and crossfire attacks [16,17], yet it is often overlooked in many models.
- Attack Target: The target of a DDoS attack is typically a server, network, or online service that the attacker aims to overwhelm or disrupt. These targets may include websites, applications, or infrastructure that are critical for an organization’s operations. The attacker floods the target with a massive volume of malicious traffic, causing it to crash or not respond. The goal is often to cause service disruption, financial loss, or reputation damage. Targets can be selected based on their prominence, vulnerability, or value to the attacker, making DDoS attacks both a tool for cybercrime and a method of protest or extortion.

Attackers use botnets and intermediary networks to launch DDoS attacks by directing malicious requests towards targeted systems. These requests traverse critical network links and intermediary servers, ultimately converging into a unified stream of attack traffic aimed at the target. This model helps explain and analyze several classic DDoS attack types, including flooding attacks [18], link flooding attacks (LFA) [17], and amplification DDoS [16].

However, when analyzing merging DDoS attack strategies, these core components are insufficient, requiring an even more sophisticated model, especially focusing on the complex phases involved.

### 2.2. DDoS Attack Chain Model

**Figure 2:**
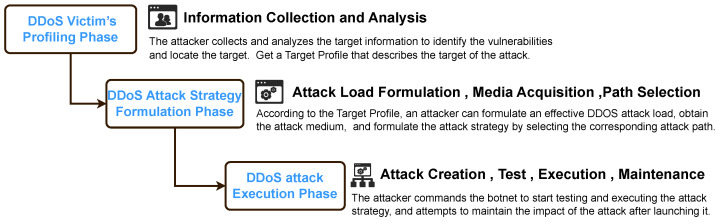
Proposed three-phase DDoS attack-chain framework. Phase I (victim profiling) produces a target profile via information collection and analysis; Phase II (strategy formulation) derives the attack strategy by attack-load formulation, media acquisition, and path selection; Phase III (execution) conducts attack creation, testing, execution, and maintenance. We use this attack-chain framework to deconstruct emerging DDoS attacks and to align defense discussion with the same phases (see Figure 4).

The DDoS attack model introduced above focuses primarily on the entities involved in the attack, which is only one part of the overall attack, but lacks a systematic description of the dynamic process of the DDoS attack and the preparatory actions by attackers. Building on previous studies [16,19], we propose a DDoS attack-chain model to structure these attacks into three distinct, consecutive phases, as shown in Figure 2. These phases are as follows:

1. Phase I: DDoS victim information collection. Before launching an attack, the attacker gathers detailed information on the victim to pinpoint their vulnerabilities. This phase involves collecting basic data about the victim target (e.g., IP addresses and open ports) through methods like phishing, port scanning, and other reconnaissance tools. Attackers often employ stealthy probing techniques, sending probing packets that mimic legitimate traffic (e.g., standard TCP requests), then analyzing the target’s responses to infer the victim’s details. For example, the Shrew attack leverages such probes to study retransmission timeout (RTO) mechanisms on network links. Through this probing, attackers pinpoint optimal attack timing, vulnerabilities, and weak points within the target’s infrastructures (e.g., misconfigured servers for amplification DDoS attacks). This way, attackers can initially define their objectives, whether to overwhelm network resources, disrupt services, or inflict reputational harm. As a result, this phase ends with the creation of a comprehensive profile of the victim, which serves as the prerequisite for the next phase.
2. Phase II: DDoS attack strategy formulation. Building on the victim profile, attackers focus on developing an effective attack strategy through three key steps. Firstly, attackers design an attack payload to exploit specific vulnerabilities. For example, payloads may vary in length to target weaknesses in regex processing [20]. Subsequently, attackers will determine the most effective delivery path for their payload. Amplification attacks, for instance, abuse servers with high response-to-request ratios to reflect attack payloads, while CDN-Convex attacks exploit latency variations in content delivery networks. Thirdly, attackers need to orchestrate their attack resources based on the attack payload and path. They will recruit some unsecured devices or critical network links as attack vectors (e.g., cloud-based botnet) in various ways [10,15,19,21,22]. Then, attackers will consider the combination and scale of attack vectors and the size of attack traffic in order to achieve the expected attack effect. Emerging attacks like ReDoS achieve disproportionate disruption with minimal resources compared to traditional flooding [20]. In the end, the outcome of this step is to create a comprehensive attack strategy, ready for execution in the next phase.
3. Phase III: DDoS attack execution. In the third phase, the attacker shall conduct the attack strategy developed in Phase II. However, before the execution, some preliminary tests are required to verify that the strategy can effectively impact the target [16,19]. Once its viability is confirmed, the attack will officially start on a large scale. Otherwise, adaptive adversaries will continuously refine their approach to counter defenses, even restarting from the first phase of the DDoS attack chain (e.g., MaDIoT attack). In addition, periodical DDoS attacks will be executed to prolong service disruption [23,24].

Obviously, each of these phases can be implemented through various methods. A comprehensive understanding of DDoS attacks, therefore, requires examining the entire sequence of activities in this attack chain [16]. By investigating adversarial behaviors across these phases, we can gain valuable insights into the evolving DDoS threat landscape and improve the corresponding defensive strategies. For instance, we can disrupt any one phase in the attack chain to prevent emerging DDoS.

### 2.3. Power Grid Infrastructure and Key Components

**Figure 3:**
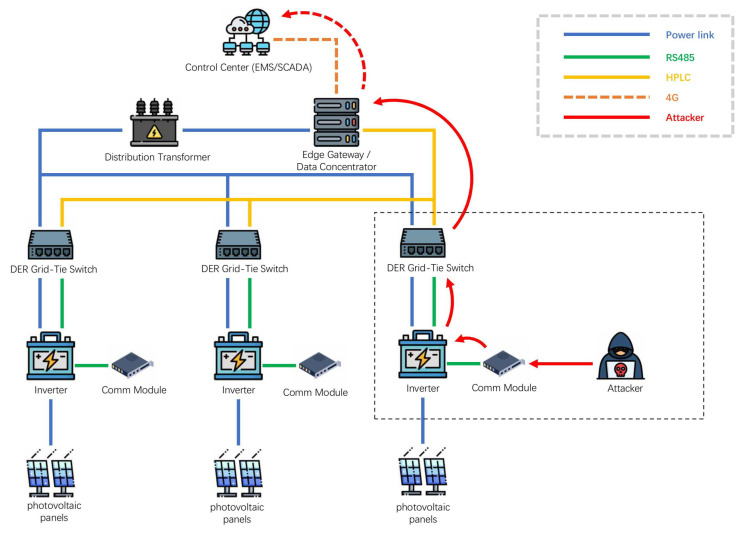
Component-level power grid cyber–physical infrastructure and operational workflow. The diagram illustrates a typical distributed energy resources (DER) setting in which a control layer (control center) communicates with field devices through heterogeneous communication media (e.g., RS485/HPLC/4G), while physical power assets (e.g., distribution transformer and DER equipment such as photovoltaic panels, inverter, and grid-tie controller) are connected via power links. The operational workflow follows a closed loop: field devices and edge gateways report measurements/telemetry upstream to the control center, and the control center issues control commands downstream to actuators that regulate power delivery and DER operation.

Building on the infrastructure in Figure 3, we relate emerging DDoS to our three-phase attack-chain framework (Figure 2) and to the exploited layers. In Phase I (profiling and timing discovery), adversaries probe exposed interfaces and communication endpoints, infer critical links and device roles, and identify high-impact operating windows (e.g., peak-load or transient conditions). In Phase II (strategy formulation), they select attack surfaces and construct coordinated plans, such as (i) flooding or intermittently congesting control/telemetry links, (ii) manipulating measurement channels along the telemetry path (e.g., FDI-style actions), or (iii) coordinating controllable edge/DER power devices to induce demand-side disturbances. In Phase III (execution), these plans are realized through sustained or bursty traffic disruption, measurement injection, and/or power-side coordination, with parameters adjusted based on observed system responses. Correspondingly, the primary exploited layers can be summarized as the communication layer, the measurement/control path, and the cyber–physical interface. This mapping also motivates phase-aligned defense choices reviewed in Section 6: network-layer active defense and redundancy to protect link availability, model-/data-driven validation and detection to protect measurement integrity, and resilient control plus physical-layer reinforcement to reduce the impact of coordinated power-side disturbances.

## 3. New DDoS Attacks Targeting Emerging Device Ecosystems

Hardware devices play a critical role in the DDoS attack model. In particular, Internet of Things (IoT) devices, which often lack robust security measures, are increasingly vulnerable to being exploited as bots in IoT-based DDoS attacks [10]. However, these devices can also be targets of device-targeted DDoS attacks. By compromising devices within a network, attackers can indirectly disrupt device-control systems’ performance. In this section, we focus on the emerging DDoS threats targeting new hardware devices.

### 3.1. IoT-Targeted DDoS Attack

The exponential growth in the number of IoT devices is a double-edged sword [10]. While they offer significant convenience, the lack of robust security measures makes IoT devices attractive targets for DDoS attackers. One notable IoT-based threat is the Mongolian DDoS, a stealthy attack characterized by the use of IoT devices as botnet nodes that conduct distributed attacks with minimal per-device traffic [25]. Its targets are IoT networks and related services, so Mongolian DDoS is an IoT-targeted DDoS attack.

Similarly, Mirsky et al. [26] introduce the concept of a 911-DDoS attack, which uses a cellphone botnet to repeatedly call emergency services, overwhelming the 911 system—a vulnerability that also affects next-generation 911 systems (NG911). Additionally, recent DDoS attacks specifically target IoT devices rather than merely using them as bots. Tushir et al. [27] reveal energy-oriented DDoS (E-DDoS) attacks against devices in Wi-Fi smart homes.

Based on the DDoS attack chain, we analyze and compare the aforementioned attacks due to the different botnets. For example, the 911-DDoS attack [26] requires a cellphone botnet, rather than Mirai [28,29] used by typical IoT-based DDoS attacks. In the attack-strategy formulation phase, these strategies resemble traditional flooding by sending large volumes of requests, yet each exhibits distinct characteristics. Both E-DDoS and Mongolian DDoS attacks rely on network request packets (e.g., ICMP). However, E-DDoS requests are crafted to trigger high-load, high-complexity computations, rapidly increasing energy consumption on the target [27]. In contrast, Mongolian DDoS attacks use smaller per-device traffic loads—typically 10–30% of normal—enabling them to evade many threshold-based detection mechanisms [25]. In 911-DDoS attacks, attackers exploit repeated emergency calls as the attack traffic. In this scenario, the botnet randomizes cellular identifiers to bypass network restrictions. Because regulations protect unidentified emergency calls, emergency centers cannot easily block these requests.

In the DDoS attack execution phase, both Mongolian DDoS and 911-DDoS attacks overwhelm target systems by gradually accumulating traffic or calls, imposing sustained load on the infrastructure. In contrast, E-DDoS attacks cause service interruption by forcing excessive energy consumption in smart-home devices. This not only disrupts service but can also lead to high electricity costs, which is undesirable for users. Even if the device’s energy is not fully depleted, the attack can still force the device to disconnect from the smart-home IoT network due to buffer overflows in the network interface [27]. This, in turn, interrupts service or significantly degrades performance.

Consequently, IoT systems require security measures that are both robust and versatile. A paradigm shift toward energy-aware detection, cross-domain correlation analysis, and regulatory-aware defense frameworks is imperative.

### 3.2. Power Attacks in ICT

In addition to E-DDoS, some research has focused on “power attacks”, which aim to overload power infrastructure in industrial control systems (ICS). Different from E-DDoS, power attacks are accomplished by manipulating the system state, misleading operations, and causing widespread outages. They can be executed in several ways:

1. False data injection (FDI) attacks: Attackers tamper with sensor measurements or data on communication links to inject incorrect values into controllers, causing misguided adjustments (e.g., false relay operation, FRO) [30,31].
2. Manipulating demand via IoT devices (MaDIoT) attacks: Attackers control large numbers of IoT devices to manipulate power demand, disrupting the balance between supply and demand [32,33,34].
3. Interfering with load-frequency control (LFC) systems (time-constrained DoS): By blocking the transmission of LFC control signals, attackers degrade the system’s ability to adjust to load fluctuations in real time [35].

Each way poses unique challenges to system stability, which can be analyzed in detail through the attack chain framework. And they vary in attack approaches and attack impact, as summarized in Table 2.

In the first attack phase, attackers must identify critical attack timing before launching the attack. Historically, a successful arbitrary power attack typically takes substantial launch time to ensure stealth. To minimize launch time, M. Jafari et al. [31] recently proposed an optimization-based formal model to compute the optimal false data injection time and injection magnitude for the optimal false data injection attack (OFDIA). The model incorporates power-system dynamics (e.g., governor droop and time constant) within an optimization framework to find the minimum time required to trigger false relay operation (FRO) across multiple generators’ dispatch cycles. An attacker selects grid load peaks, scheduled switching periods, or maintenance windows as attack times, during which abnormal data are more easily masked.

In manipulating demand via MaDIoT 2.0 [34] attacks, adversaries crawl Independent System Operator (ISO) websites and Bloomberg terminals to obtain real-time grid status and operational data. Based on this information, they compute and rank the voltage-stability index for each node, thereby identifying the grid’s weakest nodes (i.e., nodes with the smallest index). These weakest nodes indicate near-term optimal attack times.

In addition, Songlin Hu et al. [35] introduce a duration-constrained DoS attack against the communication networks of multi-area power systems by optimizing the timing model of periodic DoS campaigns. They develop an attack-parameter-dependent, time-varying Lyapunov function (TVLF) method to compute the minimum allowable dormant period and maximum allowable active period of the resilient load-frequency control (LFC) system. In this way, the attacker can determine the optimal attack timing (i.e., the active period), and the TVLF method greatly reduces prior-knowledge requirements for DoS attacks. Therefore, compared with arbitrary-timing power attacks, identifying optimal timing helps ensure efficiency and improves evasion against detection algorithms.

In the second attack phase, the above attack strategies are designed to maximize grid-frequency fluctuations by targeting the most vulnerable load nodes. The botnet in MaDIoT 2.0 typically comprises high-wattage IoT devices (e.g., EV chargers) directly connected to the grid. Given the optimal timing and weakest nodes, the attacker coordinates these devices—i.e., by turning them on or off simultaneously—to precipitate a blackout in the target grid.

In an OFDIA, attackers inject false data (e.g., load or frequency measurements) into grid communication links, misleading the control center into erroneous decisions. Falsified readings at a targeted node can make the center infer excessive or insufficient output, prompting inappropriate adjustments to generating units. The resulting mismatch between generation and load drives frequency excursions and localized overloads. Moreover, the optimal false-data magnitude in an OFDIA can be computed via optimization.

By contrast, the duration-constrained DoS strategy resembles flooding DDoS but employs intermittent bursts akin to Shrew attacks. During active periods, the botnet floods the communication network to block commands from the LFC, then pauses for predetermined dormant intervals before resuming. Unlike Shrew attacks, it imposes no constraints on rate or pulse shape, making the strategy more general. Despite differing tactics, all of the above ultimately induce grid-frequency disturbances.

In the third attack phase, attackers monitor attack effects in real time and dynamically adjust the above strategies. Because of grid safeguards, the strategies may not always trigger large or sustained frequency fluctuations. Operators restore frequency to acceptable bounds by redispatching generation toward affected nodes. Therefore, the attacker launches another attack at the next optimal time window. Ultimately, repeated cycles can induce system-frequency instability and precipitate a blackout.

As a result, the key idea behind power-grid attacks is identifying the optimal attack timing—typically when system load is highest—allowing attackers to synchronize their actions with the target’s weaknesses. Moreover, because the optimal timing changes with system status, the attack strategy can also be flexibly adjusted as the system evolves in real time. In other words, the emerging power-attack strategies above are adaptive. However, existing detection mechanisms (e.g., residual detection) are mostly based on static grid-topology assumptions and cannot effectively capture transient dynamic anomalies caused by attacks [36]. In addition, the emerging attacks described above (e.g., MaDIoT 2.0 [34]) exploit transient states (e.g., load peaks) to mask anomalies by mimicking natural fluctuations, thereby bypassing traditional threshold-based detection. Therefore, adaptive, real-time defense mechanisms are essential.

Furthermore, prior research primarily focuses on single-point FDI attack scenarios, where adversaries compromise either sensor nodes or communication links. P. Li et al. [37] propose joint-FDI attacks, in which attackers simultaneously compromise sensor nodes and the ICT network. Consequently, if the OFDIA is extended from single-point to joint attacks, its destructive power will be further enhanced. Although there are no reported real-world cases or mature studies of the joint OFDIA yet, such attacks could represent a high-risk threat in the future.

### 3.3. The Variants of Power Attack

In this section, we focus on several emerging attacks stemming from power attacks. A recent study by Shekari et al. [38] shows that a new variant of MaDIoT, named Manipulation of Market via IoT (MaMIoT), can manipulate the electricity market. MaMIoT is the first energy-market manipulation cyberattack that exploits the relationship between demand and price fluctuations. From the perspective of the DDoS attack chain, MaMIoT and MaDIoT employ similar methods, with their end goals differing. In particular, both require a high-wattage botnet to change the grid load. This requirement stems from scale: to produce a measurable aggregate-load deviation at the grid level (whether for destabilization or for market manipulation), an adversary must control a sufficiently large amount of real power that can be switched or modulated within a short time window. High-wattage IoT botnets provide this capability by coordinating many power-hungry devices (e.g., HVAC units, heaters, EV chargers, or industrial loads) so that even modest per-device changes can accumulate into a system-level load perturbation. Notably, MaMIoT and MaDIoT mainly differ in their objectives and the magnitude of perturbation—MaMIoT aims for subtle price manipulation, whereas MaDIoT seeks destabilization and outages, rather than in the underlying resource requirement. MaMIoT does not seek to cause blackouts but instead attempts to manipulate electricity prices by slightly altering total consumption. Consequently, MaMIoT can significantly increase profits for selected market participants or inflict economic damage on targets, depending on attacker objectives. In effect, MaMIoT is a subtler, less disruptive version of MaDIoT.

Exploiting optimal timing (scenario). Consider a system operating near a daily peak: the grid’s load-frequency control operates under tighter margins, and normal demand fluctuations are larger. An attacker who has identified such peak windows (e.g., via public operator/market information or by observing system dynamics) can launch a coordinated, small demand modulation using high-wattage bots. Because the perturbation coincides with naturally volatile conditions, the induced deviation can (i) achieve a higher impact-to-effort ratio and (ii) better camouflage itself as normal variability, thereby reducing the effectiveness of static threshold-based detection. This timing dependence is consistent with the observation that emerging power-attack strategies exploit transient states (e.g., load peaks) and adjust strategies as system status evolves.

Similarly, power side-channel attacks against multi-tenant data centers are variants of power attacks. Compared to traditional power attacks, they exploit novel physical side channels (e.g., voltage-side [39], thermal-side [40], or acoustic-side [41]), which leak information about benign tenants’ real-time power usage and enable attackers to determine optimal attack timing. During peak usage by benign tenants (i.e., when legitimate workloads drive higher and more variable power draw), side-channel signals are stronger, and system margins are tighter, which makes well-timed power-manipulation attempts both more effective and harder to distinguish from normal fluctuations.

In addition, power attacks can also cause devices to overheat, resulting in device outages—a threat known as a thermal attack [42,43]. Thermal attacks can use the same strategy as a power attack: manipulating a device’s power load to trigger an outage. Z. Shao et al. [44] propose a battery-assisted thermal attack. In this attack, adversaries run power-hungry applications (e.g., intensive computing) to increase a server’s power draw and heat output. The additional demand can deplete a server’s built-in battery and strain the shared cooling system in a data center, potentially causing disruption. Similarly, attackers can create localized “hot spots” in public clouds (e.g., AWS) by frequently shuffling VMs. Frequent VM shuffling (i.e., repeated placement/migration) can concentrate compute-intensive workloads onto specific hosts or racks, increasing local power density and heat output; it can also introduce additional overhead on the virtualization and network stack, further elevating resource usage. Under constrained cooling conditions, these localized hot spots may trigger thermal throttling, battery depletion in edge/colocation settings, or even device outages—thereby realizing a thermal attack whose mechanism is to manipulate power draw and heat dissipation to cause disruption. Thermal attacks also rely on identifying the optimal timing through side channels [43], much like power attacks. Therefore, thermal attacks complement power attacks.

Although we analyzed the challenges posed by power attacks in Section 3.2, the attacks discussed above further exacerbate the difficulty of defense. By exploiting new side channels, attackers can more stealthily identify optimal attack timing, making it easier to bypass defenses. Thermal attacks and MaMIoT amplify secondary impacts—such as equipment damage due to overheating and post-attack economic costs—even when primary power-attack effects are mitigated.

### 3.4. Summary of New DDoS Attack Targeting Emerging Device Ecosystems

Building on the previous review, we summarize the attack chain of emerging DDoS campaigns that target device ecosystems. This perspective reveals phase-specific characteristics of device-targeted DDoS at each stage.

- Phase I: Timing discovery and stealth. Unlike traditional DDoS, emerging attacks increasingly target device energy. To maximize stealth and impact, adversaries identify short high-activity windows—for example, peak-usage intervals of benign tenants in multi-tenant environments (where power/thermal side-channel signals are stronger) or peak-load/transient operating windows in power grids (e.g., load peaks, scheduled switching, or maintenance windows learned via operator portals). These peak/transient intervals typically exhibit larger normal variability and tighter operational margins, which can mask anomalies and increase attack effectiveness, making them attractive windows for initiation.
- Phase II: Workload manipulation and destabilization. At the chosen time, the attacker seeks to disable devices by driving workload rather than merely injecting malicious requests. Small, energy-limited devices are especially susceptible: sustained high-load tasks can drain power and destabilize control systems. Even without full depletion, continuous stress degrades stability. For large infrastructures such as the power grid, direct energy exhaustion is less effective; instead, adversaries may manipulate system state to create unbalanced loads, precipitating localized outages.
- Phase III: Periodicity and cumulative impact. Device-targeted DDoS commonly appears in periodic waves. Because device conditions vary, not every wave succeeds immediately; however, well-timed repetitions amplify overall impact and prolong downtime. The resulting interruptions can impose substantial operational and economic costs.

Optimal timing in power-grid DDoS/DoS attacks. In power-grid contexts, optimal timing refers to selecting attack start times and burst schedules that maximize disruption (or manipulation effect) while minimizing the attacker’s required resources and the defender’s detection confidence. Within our attack-chain framework, timing discovery is primarily an outcome of Phase I (profiling)—where adversaries infer peak/transient windows and weak points—and it is operationalized in Phase III (execution) through well-timed floods or intermittent bursts.

Attackers can derive timing cues from multiple channels, including publicly available operator/market information (e.g., load forecasts and price signals), observation of system dynamics (e.g., recurring peak-load patterns), and probing-based feedback (e.g., short bursts to test responsiveness). Timing is especially exploitable during peak-load or transient intervals because (i) operating margins are tighter, (ii) normal variability is higher (masking anomalies), and (iii) intermittent attack schedules can evade static thresholds. This timing dependence is consistent with the representative power attacks summarized in Table 2, where attackers explicitly identify critical windows before executing coordinated demand manipulation (MaDIoT), an OFDIA against LFC-related measurements, or intermittent, duration-constrained DoS bursts against power-system communication networks.

According to the attack chain, the key feature of device-targeted DDoS attacks is identifying the optimal attack timing, after which the attacker seeks to induce system energy overload. One driver of this feature is the differing scales of ICT systems. For example, random overload power attacks may directly cause large-scale data-center outages but cannot easily trigger large-scale power-grid failures. Such attacks are often mitigated by the grid’s resilient load-frequency control (LFC) mechanisms. Therefore, identifying optimal timing and targeting weak areas of the grid can increase attack success and stealth.

From an attack-chain perspective, emerging campaigns often combine network-level (e.g., FDI) and device-level considerations. Notably, these DDoS attacks can target not only conventional network services but also other dimensions (e.g., economic impacts caused by MaMIoT and E-DDoS). Even when attacks are detected, defender losses can far exceed the cost of the attacks. This is why integrated cross-layer defense systems are required. Moreover, limited device resources impose stricter requirements for lightweight, real-time defensive performance.

## 4. Applications of Large Language Models in Smart Grid Security

The smart grid security faces three core challenges: first, the difficulty in integrating heterogeneous data from scenarios such as IEC 61850 protocol communications and SCADA system operations, where traditional methods struggle to balance real-time responsiveness with analytical depth; second, weak generalization detection capabilities against novel cyberattacks (e.g., APT attacks, FDI attacks), with rule-based or traditional AI solutions prone to false positives and false negatives; third, in distributed device deployment scenarios, achieving a balance between privacy protection and security efficiency remains challenging. LLMs, with their semantic understanding, cross-modal integration, and generative capabilities, offer a novel technical method to address these challenges. Their main logic for empowering grid security can be summarized in four key directions: precision anomaly detection, comprehensive situation security awareness (SSA), intelligent communication security, and collaborative distributed protection. Specific application scenarios include: anomaly detection, communication security, and vulnerability detection.

### 4.1. Anomaly Detection

Traditional smart grid anomaly detection relies on manual rules or shallow machine learning and struggles to handle the diversity of complex scenarios such as GOOSE/SV message anomalies and protocol violations. By integrating domain knowledge with data-driven approaches, LLMs enhance the generalization capabilities of anomaly detection, forming two core technical paradigms.

One category is the human–machine collaborative optimization paradigm, addressing the scarcity and complexity of smart grid anomaly annotation data. Za et al. [45] pioneered the integration of an LLM with a Human-in-the-Loop (HITL) model. For four types of anomalies in digital substations—including replay attacks and data injection—they generated authentic GOOSE/SV datasets using a Hardware-in-the-Loop (HIL) platform. After training the LLM under human expert guidance, the model acquired the anomalous semantic features of smart grid communication protocols. In a three-stage training comparison, fully trained ChatGPT 4.0 achieved true positive rates of 98.18% and 96.67% for GOOSE/SV anomaly detection, validating the paradigm’s effectiveness. However, this approach relies heavily on manual annotation and inadequately addresses generalization for rare grid anomalies. To address this, Za et al. [46] further proposed the CyberGridToD system. By constructing belief vectors through task-oriented dialogue (ToD), it precisely represents system states, achieving highly reliable detection without specialized training. This overcomes the generalization shortcomings of previous approaches. However, its core limitation remains its reliance on Wireshark’s preprocessing support for protocol detail characterization.

Another category is the multimodal fusion paradigm, which overcomes the limitations of single-data-type detection. The GridSense framework proposed by Shen et al. [47] addresses the challenge of integrating multiple sources of data, such as electrical parameters, meteorological conditions, and electricity prices, into traditional SSA. Through three key modules: heterogeneous data structuring, prompt engineering optimization, and meta-learning tuning, it converts multimodal data into text prompts understandable by LLMs. This approach maintains an anomaly detection accuracy of 90.25% even in few-shot scenarios. This paradigm’s advantage lies in leveraging LLMs’ semantic integration capabilities without data restructuring. However, ablation experiments reveal insufficient recall for rare anomalies, stemming from the need to enhance the framework’s ability to distinguish core grid features from redundant information within multimodal data.

### 4.2. Communication Security

In smart-grid communications, defenders must detect and respond to abnormal communication behaviors (e.g., suspicious message patterns, protocol deviations, and coordinated disruptions) while complying with strict privacy regulations. In this context, LLMs primarily contribute as a semantic inference layer: given privacy-preserving representations of communication and system-state signals, an LLM can classify anomalies, prioritize alerts and response actions, and correlate related events across devices/regions to support timely detection and mitigation decisions. Accordingly, the following studies combine privacy-preserving data-sharing mechanisms with LLM-enabled anomaly inference. The generative AI agent framework proposed by Zeng et al. [48] employs a dual-module approach combining anonymized synthetic data generation with LLM anomaly classification. It leverages diffusion models to generate smart grid data that preserves core features while protecting privacy and utilizes an LLM for anomaly detection and response prioritization. In this pipeline, the diffusion model addresses the privacy constraint by generating anonymized yet feature-preserving training data, while the LLM provides the detection output (anomaly classification and response prioritization) that can be directly used by operators or downstream controllers to initiate mitigation workflows. On the “Smart Grid Stability” dataset, this framework demonstrated high alignment between synthetic and original data, along with fast training convergence and excellent real-time performance. The innovation lies in advancing privacy protection to the data generation stage, though it does not address collaborative anomaly detection across regional smart grids. The federated learning (FL) and LLM hybrid framework proposed by Pei et al. [49] addresses the protection needs of distributed devices (PV inverters, smart meters). Through a layered design combining lightweight detection at edge nodes with central LLM semantic inference, it transmits only model parameters rather than raw data. This approach ensures privacy while enabling root cause analysis of anomalies and threat correlation. Edge nodes focus on lightweight local detection, whereas the central LLM aggregates high-level signals (e.g., alert summaries or model updates) to perform semantic interpretation and cross-site correlation, turning distributed indicators into a coherent detection narrative and actionable mitigation priorities. While this approach aligns with the distributed deployment characteristics of smart grids, the inference latency of the central LLM still requires optimization to meet the real-time demands of industrial control systems.

Overall, the role of LLMs in communication security is not to replace traffic filtering or encryption primitives but to strengthen decision-making in detection and mitigation: (i) interpreting heterogeneous communication/security signals, (ii) producing anomaly labels with contextual explanations, and (iii) prioritizing responses under privacy-preserving data-sharing constraints.

### 4.3. Vulnerability Detection

Detecting inherent vulnerabilities in smart grid industrial protocols (Modbus, IEC 61850) relies on fuzz testing, but traditional methods suffer from low test case effectiveness, difficult log parsing, and non-interpretable results. Aldysty et al. integrated LLMs into the entire fuzz testing workflow [50], leveraging their deep understanding of protocol syntax and semantics to generate highly compliant and diverse test cases (GPT-4o achieved a 99.46% test case acceptance rate, while the Mistral-Smal model only reached 25.62%). This simultaneously enhanced testing transparency by optimizing log parsing and vulnerability explanations through an LLM while mitigating LLM hallucinations via Retrieval-Augmented Generation (RAG).

## 5. The Synergistic Effect of Large Models and Deception Defense

During the intelligent, digital, and industrialized upgrade of new power systems, automated intelligent attacks based on LLMs have become increasingly frequent, posing more severe security challenges to smart grid [51]. Concurrently, the previously described proactive defense theories relying on deception techniques (such as honeypot systems) exhibit three major pain points: (i) Honeypot logs contain mixed formats of industrial protocol data and commands, making manual parsing inefficient and delaying threat intelligence extraction. (ii) Static honeypots have fixed response patterns, making them easily identifiable by intelligent attack tools and lacking sufficient interaction realism. (iii) Deception data (e.g., device configuration files and protocol packet decoys) must adapt to diverse grid equipment, making large-scale generation challenging. The integration of LLMs with deception defense fundamentally enhances intelligence extraction through semantic understanding, adapts responses to improve interaction realism, and enables scalable data production via modular design.

### 5.1. Threat Intelligence Extraction: From Manual Parsing to Intelligent Parsing

The industrial characteristics of smart grid honeypot logs (multi-protocol, strong device correlation) necessitate log parsing that balances “protocol precision” and “attack intent identification.” Large Language Models (LLMs) achieve breakthroughs through two technical approaches. The direct parsing approach focuses on lightweight deployment, suitable for edge grid nodes. The GPT-2C system proposed by Setianto et al. [52] transforms honeypot log parsing into a span-based question-answering task. By fine-tuning the GPT-2 model to adapt to Unix command features in grid SSH honeypots, it achieves an F1 score of 0.89 on the CyberLab dataset. Training completes in just 2 epochs, with inference latency meeting real-time requirements. This approach’s core advantage lies in low deployment costs, but it struggles with parsing industrial protocol commands like IEC 61850, requiring additional protocol preprocessing modules.

The Retrieval-Enhanced Approach addresses command obfuscation and slow extraction of attackers’ Tactics, Techniques, and Procedures (TTPs) from massive logs of highly interactive honeypots. Lanka et al. [53] designed a framework centered on GPT-4-turbo, integrating Bashlex for command normalization and cosine similarity matching to identify malicious features. Validation in AWS/GCP environments achieved a session clustering silhouette coefficient of 0.98 and an F1 score of 0.91, significantly reducing threat response times. Similarly, Ozkok et al. [54] employed GPT-4 to parse Elasticsearch and SSH honeypot logs, achieving mapping to the MITRE ATT&CK framework. However, confusion and false positives persist, primarily due to the incomplete knowledge base of attack signatures specific to the smart grid.

To provide a cross-study comparison, Table 3 summarizes representative LLM-assisted approaches for threat-intelligence extraction from honeypot data, including the datasets used, detection methods, accuracy, the LLM selected, and response time (when reported).

### 5.2. Dynamic Interactive Simulation: From Static Response to Adaptive Deception

The realism of honeypot interactions directly determines deception effectiveness. LLMs balance “realism” and “deployment feasibility” through different technical approaches, forming three distinct solution categories.

Commercial LLM-driven solutions prioritize high realism, leveraging pre-trained data for rapid scenario adaptation. Sladic et al. [55] propose a shelLM system, built on GPT-3.5-turbo-16k, that employs personality prompts and Chain of Thought (CoT) guidance alongside session state persistence mechanisms. It achieved a 90% true negative rate across 226 command tests, effectively deceiving human attackers. Ragsdale et al.’s FEI framework reduces token consumption through dynamic context management [56]. However, both approaches face compliance risks due to the potential leakage of sensitive smart grid data and incur high long-term operational costs, making them more suitable for laboratory-level threat analysis. Open-source LLM fine-tuning solutions prioritize autonomy and adaptability for grid edge deployment. Otal et al. [57] built a dataset containing Cowrie attack commands and Linux command manuals based on Llama3 8B. Through quantization optimization using SFT, LoRA, and QLoRA, they achieved a cosine similarity of 0.695 across 140 samples, accurately simulating Linux command responses (including error reporting for invalid commands). Christli et al. [58] designed an open-source LLM honeypot, though untuned, and optimized deployment via an SDLC waterfall model. Its core limitation lies in response realism being dependent on training data quality, with insufficient simulation capability for smart grid industrial protocols.

Hybrid architecture solutions balance effectiveness and cost, making them the preferred choice for smart grid scenarios. Guan et al.’s HoneyLLM [59] filters scripted attacks at the frontend while processing valid sessions with a backend LLM. Results show GPT-4o-supported average session length reached 5.83 rounds, far exceeding Cowrie’s 2.96 rounds. Yang et al.’s ShellBox [60] further addresses multi-turn consistency through dynamic error simulation (81.63% prompt injection defense accuracy) and interaction history pruning (34.5% TCS improvement). However, the core challenge for such approaches remains aligning LLM response latency with the real-time interaction demands of the smart grid.

### 5.3. From Manual Configuration to Automated, Scalable Deception Data Generation

Traditional spoofing data generation relies on manual methods or specialized tools, making it difficult to adapt to the diversity of smart grid equipment. LLMs have achieved a breakthrough through modular design. Reti et al.’s approach [61] innovatively proposes 16 prompt building blocks to generate seven categories of deception data (e.g., robots.txt, honeypots, and configuration files). The optimal prompt generated a robots.txt file with an 8.71 consistency score against real website samples. GPT-3.5-generated honeypots achieved only a 15.15% detection rate, significantly outperforming traditional methods. The core value of this approach lies in its “full-scenario adaptability”, enabling both honeypot filling and production system deployment. However, its accuracy in generating grid-specific data formats like IEC 61850 configuration files remains insufficient, necessitating prompt engineering optimization through integration with grid protocol knowledge bases.

## 6. Defense Mechanisms Against Emerging DDoS Attacks

**Figure 4:**
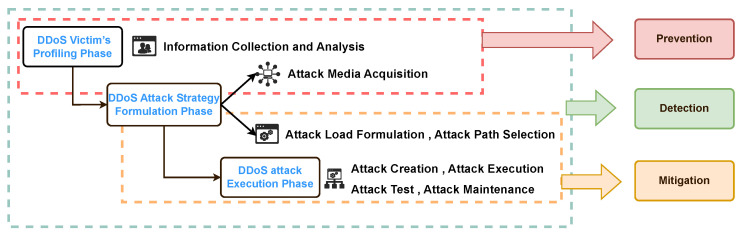
Phase-aligned defense view corresponding to the proposed three-phase attack chain (Figure 2). Prevention and early detection aim to disrupt Phase I (profiling) and Phase II (strategy formulation), while detection and mitigation mechanisms are critical in Phase III (execution) to reduce attack impact and maintain service availability. This figure summarizes how the defense mechanisms reviewed in Section 6 map to different phases of the attack chain.

Building on the attack-chain analysis above, we elucidate the lifecycle of new DDoS attacks. The increased stealth and sophistication of modern DDoS campaigns make them difficult to defend against; however, disrupting any single phase of the attack chain can minimize their impact. Hence, defenses should align with the same three phases (Figure 4). We next review existing defense mechanisms against the new DDoS attacks discussed above.

### 6.1. Defending Against DDoS Attacks on New Hardware Devices

As discussed in Section 3, new hardware devices not only serve as attack vectors in DDoS campaigns (e.g., botnets, amplifiers) but also become direct targets of emerging device-focused attacks. From a device-centric perspective, the most direct approach is to disrupt Phases I and II of the DDoS attack chain. In particular, whether device-based or device-targeted, attacks can be prevented by identifying and isolating compromised hardware. However, these measures are not always sufficient; DDoS detection and mitigation remain essential components of defense.

#### 6.1.1. Enhancing Detection of DDoS Attack Infrastructure

Botnets consisting of IoT devices have become a major source of DDoS attacks. Therefore, the most fundamental way to prevent DDoS is to prevent devices from becoming bots. To mitigate 911-DDoS, antivirus (AV) scanners on smartphones can block downloaded IoT-botnet malware (e.g., trojans) [26]. Emergency services can also disallow NSI calls or perform whitelisting with trusted device identification. Moreover, Amir Javadpour et al. [62] propose an SDN-oriented, cost-effective edge-based MTD approach (SCEMA). They argue that hosts connected to more critical servers are prime infection targets; accordingly, SCEMA uses the number of connections between hosts and critical servers as a key feature and reduces exposure by shuffling the most-connected hosts.

Moreover, enhanced detection of botnets can help prevent the generation of device-based DDoS attack traffic. Borges et al. [63] propose a time-series anomaly-detection method using multiscale ordinal patterns and Isolation Forest, achieving 99.5–100% accuracy in identifying botnets by monitoring packet-transmission dynamics. This approach leverages IoT devices’ predictable operational patterns, where deviations signal compromise. Another method uses the *message innovation rate* (MIR) to distinguish legitimate users from botnet nodes [64]. MIR measures the novelty of messages (e.g., HTTP requests) sent in a network; legitimate users typically exhibit higher MIR due to greater request diversity. Therefore, although individual bot requests may appear normal, their lower innovation rate betrays coordinated behavior, enabling systems to cluster similar message patterns and disable the bots.

To prevent Mongolian DDoS attacks, the online discrepancy test (ODIT) [25], a nonparametric anomaly-detection algorithm, monitors packet rates and identifies deviations from normal behavior. Once an attack is detected, the system analyzes which devices are the sources and then blocks traffic from those devices.

In addition, as depicted in Figure 1, many devices in intermediate networks can be leveraged for DDoS attacks. For example, DNS tunnels—valued for their versatility and concealment—are a preferred method for executing command and control (C&C) [65]. The GraphTunnel framework provides a GNN-based defense for detecting DNS tunnels and identifying tunneling tools. It constructs DNS recursive-resolution graphs that represent the resolution process as graph paths; these paths are analyzed with GraphSAGE to aggregate features from neighboring nodes, enabling effective detection even in complex scenarios.

Beyond tunneling, prior research [66] identifies millions of publicly reachable hosts and various middleboxes [67] that can be abused as amplifiers for amplification DDoS. To evaluate vulnerability, Christian Rossow et al. [66] define the bandwidth amplification factor (BAF) and packet amplification factor (PAF) to measure attack impact. Building on BAF and PAF, Johannes Krupp et al. [68] present *AMPFUZZ*, the first systematic, protocol-agnostic approach to discovering amplification vectors (i.e., amplifiers) in UDP services. AMPFUZZ employs state-of-the-art greybox fuzzing enhanced with a UDP-aware technique, enabling effective amplifier discovery.

To estimate amplification risk on servers more accurately, Moon et al. [69] developed *AmpMap*, a measurement framework that identifies novel amplification patterns missed by traditional methods. AmpMap deeply scans and analyzes query patterns across protocols and servers to determine which requests trigger amplification and to quantify amplification factors (AFs). For middlebox-based amplification attacks, restricting injection conditions, limiting injected-response sizes, or disabling HTTP injection altogether can prevent abuse [67].

*Pros and Cons:* Enhancing security on the device side can reduce major sources of DDoS traffic and prevent a large share of attacks. However, these technologies alone cannot fully contain DDoS campaigns. Owing to equipment heterogeneity, it is difficult to apply a single technique uniformly to detect bots and amplifiers. SCEMA, an MTD-based approach, lowers exposure, but frequent shuffling and topology changes may introduce detection latency and additional resource overhead.

Moreover, most methods above rely on machine learning or specific network architectures (e.g., SDN) rather than device-resident controls. Although many report high accuracy, deployment complexity, detection latency, scalability limits, and manual threshold tuning constrain their practical utility. Therefore, attack-specific detection and mitigation remain indispensable. In practice, these limitations often stem from (i) the need for additional instrumentation or controller-side modifications to deploy the defense at scale, (ii) the delay introduced by watermark injection/estimation or by collecting sufficient evidence for statistical tests, (iii) the difficulty of maintaining consistent performance across heterogeneous devices and operating conditions, and (iv) the sensitivity of threshold-based detectors to time-varying traffic/load baselines, which can increase false alarms or missed detections unless thresholds are continuously retuned.

#### 6.1.2. Detection Mechanisms for False Data Injection Attacks

Several methods have been developed to detect FDI attacks, which can be broadly divided into model-based and data-driven detection methods. Model-based methods detect inconsistencies between actual measurements and model-predicted (estimated) measurements derived from an accurate physical-system model, typically by monitoring the resulting residual signals. Dynamic watermarking is a representative technique that injects small, controlled perturbations into system parameters or control inputs so that the predicted response becomes verifiable; deviations from the expected “watermarked” behavior can then be used to statistically flag stealthy injections.

Higgins et al. [70] propose a stealthy moving-target defense (MTD) that injects Gaussian-noise watermarks into line admittances to prevent traditional MTDs from being bypassed by unsupervised-learning-based attacks. The method integrates cumulative-sum (CUSUM) error detection, achieving near-100% attack-detection rates with low perturbation (1%) [70], though a fixed threshold limits robustness under dynamic loads. Xu et al. [71] advance robust MTD by optimizing admittance perturbations via Jacobian-subspace decomposition, ensuring worst-case detection against unknown attacks. Additionally, Liu et al. [72] propose a recursive watermarking (RWM) method combined with an unknown-input observer (UIO), i.e., an observer that estimates system states while attenuating the influence of unmeasured disturbances or unknown inputs. This design enhances detection by jointly optimizing watermark strength, UIO parameters, and control gain to improve residual sensitivity to injected data while maintaining closed-loop control performance.

In contrast, data-driven methods are more flexible. They leverage machine learning and signal processing to capture attack patterns dynamically in complex scenarios. Recently, the massive volume of data collected by Advanced Metering Infrastructure (AMI) has accelerated the application of machine learning (ML) in data-driven detection [73]. However, smart-grid measurement datasets typically exhibit skewed class distributions and partial labels due to high labeling costs, which degrades the detection performance of data-driven methods.

To address this, Miao et al. [74] propose the DeSSW framework, which combines an optimal-transport (OT) reweighting algorithm with a debiased self-training strategy. DeSSW leverages self-training to exploit plentiful unlabeled measurements, learning separable representations for normal and attacked measurements in feature space and thereby improving FDI identification.

To further cope with data privacy and communication costs, Tran et al. [36] propose a two-layer-encrypted federated-learning framework that trains a global FDI detector while preserving cross-agency data privacy, using lightweight encryption under the DCR assumption and dynamic key management.

Unlike ML schemes that rely on large, high-quality training sets, the redundant sparse reconstruction (RSR) algorithm [75] requires only real-time measurements for attack localization and signal recovery. Using a redundant mapping matrix and random scrambling with a chaotic system, RSR generates two distinct reconstructions of system states to detect and mitigate FDI attacks. Compared with most learning models, the RSR algorithm [75] takes about 0.04 s per detection, yielding lower latency.

In addition, a novel data-driven framework, ORIGIN, is proposed [76]; it consists of three modules: detection, classification, and signal recovery. The detection module performs least-squares fitting on the trajectory of the phasor measurement unit (PMU) signal in the complex plane via a sliding window and computes its distance from the origin to flag attacks. The classification module uses the an ensemble classifier (e.g., ICON) ensemble to distinguish FDI attack types from detected samples, and the recovery module dynamically removes injected spurious data. However, the framework’s performance depends heavily on PMU data quality. Moreover, because it does not exploit physical grid information (e.g., topological constraints) for consistency checks, it may yield false positives or missed detections. Consequently, data quality sets the upper bound on the effectiveness of data-driven methods.

*Pros and Cons:* Compared with model-based methods, data-driven methods are better able to adapt to evolving attack strategies and detect complex attacks. By contrast, model-based methods are more real-time without large training sets. However, none of the defenses above currently mitigates the OFDIA. In practice, an OFDIA can bypass both physical-validation checks (e.g., sparse legitimate-looking injections) and data-driven models (e.g., adversarial perturbations). While Jafari et al. [31] show that securing additional load meters can help, a novel multilayer framework remains essential.

Hybrid architectures that combine physical and data-driven methods warrant further exploration. For example, model-based methods can enhance data quality for frameworks such as ORIGIN [76]. Defenders must carefully balance detection accuracy against real-time performance when deploying such hybrids.

#### 6.1.3. Defending Against Emerging Power Attacks in the Power Grid

Drawing on existing research, we review defenses against power-grid DDoS along three dimensions: control-layer optimization, network-layer active defense, and physical-layer reinforcement.

We relate these three defense dimensions to our three-phase attack chain. In Phase I (profiling/timing discovery), network-layer monitoring and anomaly intelligence can reduce the attacker’s ability to learn stable operational patterns, while physical-layer protections can reduce information leakage and narrow exploitable operating windows. In Phase II (strategy formulation), network-layer active defense (e.g., segmentation, access control, and adaptive filtering) can constrain reconnaissance-to-orchestration pathways, and control-layer design can limit the attacker’s effective degrees of freedom by reducing sensitivity to timing-optimized perturbations. In Phase III (execution), physical-layer reinforcement and resilient control mitigate the physical consequences of coordinated disturbances, and network-layer mechanisms sustain the delivery of critical monitoring/control traffic under DoS/DDoS conditions. We therefore discuss each layer by stating its primary objective across Phases I–III, followed by representative mechanisms and limitations.

Physical-layer reinforcement is the most common DDoS defense in power grids. These methods primarily block attack paths through hardware design or infrastructure modification. Under-frequency load shedding (UFLS) and under-voltage load shedding (UVLS) address disturbances from natural load variation and contingencies such as outages [32]. MaDIoT 1.0 attacks can be mitigated in this way. However, such schemes are less effective because distinguishing natural load variation from MaDIoT 2.0 activity is difficult. Shekari et al. [34] propose tuning these schemes to first shed load at nodes exhibiting markedly faster voltage drop than others—locations likely under MaDIoT 2.0 attack. This adaptive scheme reduces load in the attack area and helps the system recover gradually.

In addition, mitigation against power side-channel attacks—e.g., integrated voltage regulators (IVRs), voltage-noise injection, and encapsulated/on-package decoupling capacitors (OPDs)—has shown effectiveness in data centers [77]. Although effective, these methods often lack flexibility and scalability.

At the control layer, defenses enhance inherent system stability through improved controller design. A recent study [78] proposes an elastic load-frequency control (LFC) strategy under attack-time constraints. Using an attack-parameter-dependent Lyapunov function, the controller leverages statistics of attack duration to accelerate frequency recovery during attacks. Nevertheless, these methods rely on detailed topology and attack-parameter information, which is difficult to obtain in complex smart grids.

At the network layer, defenses use machine learning or software-defined networking (SDN)-based event-triggered mechanisms such as ISETM to detect and block attack traffic. One study [79] proposes an Interval-Secure Event-Triggered Mechanism (ISETM) that actively guarantees transmission of control instructions via SDN’s dynamic defense capabilities, addressing event-triggered control (ETC) failures under attack. Additionally, a practical countermeasure against MaMIoT attacks is to deploy non-intrusive load monitoring (NILM) or non-intrusive appliance load monitoring (NIALM) algorithms on household meters in the grid [38]. These algorithms analyze voltage and current at the meter to infer energy consumption; with deep learning, reliable NILM can detect MaMIoT activity quickly and notify homeowners and utilities.

The defense mechanism at the physical layer can play a certain preventive role by optimizing the infrastructure or system. Nevertheless, these methods heavily rely on specific information about aggressive behavior and model parameters, while collecting such information is extremely challenging in high-complexity smart grids. Moreover, equipment inconsistencies, deployment difficulties, and complex grid scenarios limit the performance and practicability of these methods. In contrast, methods at the network layer are more flexible and more suitable for complex power grid scenarios. However, none of them can independently deal with dynamic, cross-layer coordinated attacks (such as the combination of MaMIOT and MaDIoT attacks). Future research could explore cross-layer cooperative defense, such as combining the elastic design of the control layer with noise injection in the physical layer to deal with complex attack scenarios.

## 7. Insights and Lessons Learned

In this section, we discuss insights and lessons learned from the extensive survey conducted in this work. Based on our discussion, we want to predict how DDoS attacks are likely to evolve within emerging network environments. Additionally, we outline the essential characteristics that future defensive solutions will need to effectively counter these evolving threats.

The landscape of Distributed Denial-of-Service (DDoS) attacks is evolving in response to advancements in technology. By analyzing emerging trends, we identify several key characteristics of these new attack strategies:

- Diversification of Attack Targets: Emerging DDoS attacks are no longer limited to traditional network services. They now target a broader range of systems, including critical non-cyber domains such as energy systems, hardware devices, and even market mechanisms. It is worrisome that these targets are often less protected than traditional network infrastructure. As a result, they can be easier to compromise, and attackers can indirectly cause service disruptions by striking these adjacent targets.
- More Cost-Effective Attack Strategies: A significant shift in emerging DDoS is the emphasis on cost-effective methods—the perennial goal is maximum disruption with minimal resources. Attacks such as MaDIoT 2.0 [34], ReDoS [80], and other asymmetric application-layer DDoS (AL-DDoS) campaigns [3] show how smaller, computationally lighter botnets (lower attack cost) can achieve impacts comparable to larger, more resource-intensive floods. These strategies often rely on advanced reconnaissance using legitimate traffic to infer system configurations—activity that victims cannot easily prevent. By analyzing responses to probes, adversaries can compute the minimum necessary traffic and optimal timing to maximize impact while reducing detection risk. Although these calculations can be complex and require prior knowledge, their payoff is high (e.g., MaDIoT 2.0 [34]), enabling disruption with fewer resources.
- More Adaptive and Intelligent Tactics: Artificial intelligence (AI) may play an important role in emerging attack strategies. While there are no confirmed reports of AI-driven DDoS, AI could automate probing, attack-strategy formulation, and execution. By leveraging feedback, attackers could rapidly adapt to changing conditions, tune parameters, and fine-grain tactics for optimal effect. Such automation lowers the skill threshold and broadens adversary access. Moreover, intelligent orchestration enables rapid shifts in attack types and combinations, predicting effects based on observed defenses and launching increasingly sophisticated campaigns—an evolutionary advance in DDoS threats.

DDoS attacks will continue to evolve with advances in AI, machine learning, and other emerging technologies. These campaigns will likely become more intelligent, adaptive, and harder to detect and mitigate, posing significant challenges for defenders. To address these threats, future defenses should integrate adaptive monitoring, detection, and response, leveraging AI to anticipate and counter new tactics and campaigns.

### Future Research Directions

Due to attacks constantly evolving, defense mechanisms must evolve as well. So we suggest the following research directions.

1. Collaborative defense mechanism: In the face of larger-scale DDoS attacks, both organizations and individuals are particularly vulnerable when fighting a DDoS alone. Adaptive defense mechanisms and cross-domain collaboration defense mechanisms will be important directions for future research. By spreading attack traffic across various nodes, multi-nodes maximize each network’s ability to mitigate large-scale attacks, thereby reducing the risk of a single point of failure [4,81]. This approach will enhance overall network performance and security. However, how to design a reasonable incentive mechanism and spreading strategy is a critical problem for researchers. They also need to be concerned about the problem caused by Yo-Yo attacks: attackers can disrupt the collaborative mechanism by periodically launching various attacks, inducing these systems to mobilize system resources repeatedly [23]. In the future, this problem may become a hidden danger.
2. Broader detection mechanisms: A capacity of broadly detecting more attacks is indeed what the current DDoS detection mechanisms are pursuing. Deploying a unique detection mechanism for each attack has low flexibility and scalability and brings enormous economic costs and resource occupation. In particular, current detection methods for Redos attacks often require special design and are not suitable for other AL-DDoS attacks [80]. Therefore, broader detection mechanisms implemented in a more general way will become mainstream in the future.
3. Sufficient robustness to intelligent attacks: As we discussed above, an attacker may use adaptive tactics to make defense strategies ineffective. Admittedly, there have been no reports of AI-driven attacks yet. It is enough to get our attention, and we need to proactively update our cybersecurity measures. AI-driven attacks may make automatic detection and blocking more challenging. In turn, we can also apply AI methods to enhance our defense system.
4. High-cost-effectiveness defense mechanisms: An advanced system should have a cost effective advantage in the game of attackers and defenders. In other words, when faced with a DDoS attack, even if the defender successfully defends against the attacker’s failure to perform a denial of service to the target, if the resource consumption (both monetary and computational) of the defender’s mobilization is greater than the attacker’s, this can also be regarded as an effective attack in a sense to the attacker. Therefore, an excellent defense system should reduce the cost of organizing the defense. There are currently two mainstream cost reduction approaches: (i) improving resource organization efficiency and (ii) reducing equipment costs. The former is represented by various network-level resource scheduling schemes, which are dedicated to finding the optimization of DDoS defense costs at the social level. The latter is currently represented by various defense systems based on programmable switches. We have noticed that many collaboration schemes take business ideas and human nature into consideration and design mechanisms to increase the enthusiasm for participating in the defense of the entire network.

## 8. Conclusions

This survey characterizes emerging DDoS threats using an attack-chain perspective that separates operations into preparation, development, and execution. This framing makes explicit the dependencies among resource acquisition, weaponization/orchestration, and multi-vector delivery, complementing prior work that largely emphasizes the execution phase. We demonstrate that modern DDoS extends beyond volumetric floods to protocol- and application-layer exhaustion and increasingly adaptive evasion, while SDN, blockchain, and heterogeneous IoT/OT deployments expand the attack surface. Consequently, defenses should target multiple points along the attack chain, integrating multi-layer telemetry, automated response orchestration, and adversarially robust learning under operational constraints. Finally, we identify LLM-enabled opportunities for scalable threat-intelligence analysis, improved anomaly detection and situational awareness, and stronger deception (e.g., higher-fidelity interactive honeypots and consistent decoy artifacts). Future research should prioritize cross-domain coordination and incentives, broader and more generalizable detection, robustness to adaptive adversaries, and cost-effective, deployable protection.

## Figures and Tables

**Table 1:** Comparison with the existing surveys on the topics of DDoS attacks.

Ref	Year	Topic Discussed	Analysis of Traditional DDoS Attacks	Analysis of New DDoS Attacks			DDoS Defense Mechanisms
				New DDoS Attacks Against New Hardware Devices	New DDoS Attacks Against New Network Architecture	New DDoS Attacks at the Application Layer	
[5]	2019	Defense Mechanisms Against DDoS Attacks in a Cloud Computing Environment	Yes	No	Yes (Only New DDoS Attacks in Cloud Computing)	No	Yes (Only Defense Mechanisms of Cloud-based DDoS Attacks)
[6]	2019	DDoS Attacks at the Application Layers	No	No	No	Yes (Only DDoS Attacks at the Application layer)	Yes (Only Defense Mechanisms of Application-Layer DDoS Attacks)
[7]	2021	DDoS Defense Solutions in SDN	No	No	Yes (Only New DDoS Attacks in SDN)	No	Yes (Only Defense Mechanisms of SDN-based DDoS Attacks)
[8]	2023	Fuzzy Logic-Based DDoS Attacks and Network Traffic Anomaly Detection Methods	Yes	No	No	No	Yes (Only Anomaly Detection Methods of Traditional DDoS Attacks)
[9]	2023	The Current State of the Art on DDoS Attack Prediction	Yes	No	No	No	Yes (Only Prediction Methods of Traditional DDoS Attacks)
[10]	2023	DDoS Attacks Over IoT Networks and Their Countermeasures	Yes	No	No	No	Yes (Only Defense Mechanisms of Traditional DDoS Attacks)
[11]	2023	DDoS Attacks in Industrial IoT	Yes	No	No	No	Yes (Only Defense Mechanisms of Traditional DDoS Attacks)
[12]	2023	A Comprehensive Survey on DDoS Defense Systems	Yes	Yes (Limited)	Yes (Only New DDoS Attacks in Cloud Computing and SDN)	Yes (Only New DDoS Attacks Based on HTTP 2.0)	Yes (Analysis of Emerging Defense Method of DDoS Attacks from Multi-dimension)
This work	2026	Emerging DDoS Attacks and Defenses With a Three-Phase Attack-Chain Framework	Yes	Yes	Yes	Yes	Yes (Phase-Aligned Defenses); Includes LLM-Enabled and Deception-Based Perspectives

**Table 2:** Comparison of power attacks in ICT.

Attack Type	Manipulating Demand via IoT Devices Attack 2.0 (MaDIoT 2.0)	Optimal False Data Injection Attack (OFDIA)	Duration-Constrained-Only DoS Attack
Attack Target	Power system high-wattage IoT devices	Load-Frequency Control (LFC) of the power system	Communication network of the power system
Botnet	Botnet composed of high-wattage IoT devices	Botnet composed of compromised load measurement and communication devices	Botnet composed of compromised networked devices (e.g., computers, routers, IoT devices)
Methods to Identify Attack Timing	Crawling ISO website and Bloomberg terminals, calculating the voltage stability index of each node	Analyzing system load and frequency data, selecting high-load periods and weak points	Calculating by an attack-parameter-dependent time-varying Lyapunov function (TVLF) approach
Attack Strategies	Manipulating high-wattage IoT devices to create power grid instability	Injecting false load, generation, or frequency data through network intrusion and data tampering	Intermittent sending of large volumes of fake traffic to block or disrupt the communication network
Main Impact	Causes load fluctuations and frequency fluctuations	Causes frequency fluctuations	Causes communication disruption and frequency fluctuations

**Table 3:** Compact comparison of representative LLM-assisted honeypot-log analysis studies.

Ref	Datasets Used	Detection Methods	Accuracy	LLM	Time
[52]	Cowrie SSH logs; Unix-command corpus	GPT-2 fine-tune; span-QA parsing	F1 = 0.89; 89% infer.	GPT-2	Train 2:54; less than 4 s (1/100)
[53]	SSH honeypot sessions (7 days)	K-means + embeddings; GPT-assisted analysis	Sil = 0.98; F1 = 0.91	GPT-4-turbo	NR
[54]	Elasticsearch (627 req.); SSH (73 attacks)	Prompted LLM: explain + MITRE map	Explain: 96.65%/97.26%; MITRE: 72.46%/98.84%	GPT-4	NR

## Data Availability

Data sharing is not applicable to this article as no new data were created or analyzed in this study.

## Abbreviations

The following abbreviations are used in this manuscript:

DDoS	Distributed Denial-of-Service
APTs	Advanced Persistent Threats
DNS	Domain Name System
LLMs	Large Language Models
KC	Kill Chain
